# Supplementary material for: Deep learning-based image enhancement in optical coherence tomography by exploiting interference fringe
Source: Commun Biol. 2023 Apr 28;6:464. doi: 10.1038/s42003-023-04846-7 (PMC10147647; doi:10.1038/s42003-023-04846-7)
Supplement: Supplementary file 2 — Supplementary Information [file 42003_2023_4846_MOESM2_ESM.pdf]

Supplementary Information

Deep learning-based image enhancement in optical coherence tomography by exploiting interference fringe

Woojin Lee[1], Hyeong Soo Nam[1], Jae Yeon Seok[2], Wang-yuhl Oh[1], Jin Won Kim[3], and Hongki Yoo[1, *]

[1]Department of Mechanical Engineering, KAIST, Daejeon, 34141, Republic of Korea

[2] Department of Pathology, Yongin Severance Hospital, Yonsei University College of Medicine, Yongin, Gyeonggi-do, Republic of Korea

[3]Multimodal Imaging and Theranostic Lab, Cardiovascular Center, Korea University Guro Hospital, 80, Guro-dong, Guro-gu, Seoul 152-703, Republic of Korea

[*]Correspondence: h.yoo@kaist.ac.kr

Supplementary Note 1

Quantitative metrics

The performance of the proposed deep learning-based OCT image enhancement framework was quantitatively evaluated using six evaluation metrics: mean square error (MSE), structural similarity index (SSIM), multi-scale SSIM (MS-SSIM), peak signal-to-noise ratio (PSNR), beta parameter (β), and edge preservation factor (EPF). The first three metrics (MSE, SSIM, MS-SSIM) compare how similar the input and the output are to the ground truth, and are used to verify the individual performances of each trained model. The other metrics (PSNR, β, EPF) were used to investigate how much further enhancement can be achieved with preservation of spatial feature information when the ground truth-level data, the best image currently available, are entered.

MSE is defined as:

$$MSE = \frac{1}{N}\sum(I_{out} - I_{ground\ truth})^2 \tag{S1}$$

where I_{out} and $I_{ground\ truth}$ are generated images and ground truth images, respectively, and N is total number of pixels in images. MSE is a criterion for pixel-wise comparing of the two images to measure similarity. A smaller value indicates more similarity at the pixel level.

As elaborated in the loss function, SSIM is a normalized metric that measures the structural similarity of two images; it is defined as:

$$SSIM(x,y) = \frac{(2\mu_x\mu_x + C_1)(2\sigma_x\sigma_x + C_2)(\sigma_{xy} + C_3)}{(\mu_x^2 + \mu_y^2 + C_1)(\sigma_x^2 + \sigma_y^2 + C_2)(\sigma_x\sigma_y + C_3)} \tag{S2}$$

where σ, μ are the standard deviation, mean operator, and C_n is a pre-determined constant. Specifically, SSIM can compare the brightness, contrast, and structure of two images.

MS-SSIM is similar to SSIM, but it produces more global similarity by measuring at multi-scale. The overall calculation process is the same, but each SSIM value is obtained in multi-scale and summed by weighting. SSIM and MS-SSIM can takes values between 0 and 1 (where 1 represents an image that is identical to the ground truth).

PSNR was used to quantify the signal strength compared to the noise level in an enhanced OCT image compared to an original image, in a process defined as follows:

$$PSNR(I_{out}, I_{input}) = 10log_{10}\frac{\max(I_{out})^2}{MSE(I_{out}, I_{input})} \tag{S3}$$

β is used to determine the degree of smoothing in the denoised images. This metric is essentially a correlation coefficient that quantifies how well the denoised image preserves the morphological features of the original image while reducing the noise. A β value close to unity indicates that edge sharpness is well preserved. The β value is also widely used as a performance measure indicator in OCT studies that statistically reduce speckle noise [1,2]; it is defined as follows:

$$\beta = \frac{\Gamma(I_{out} - \mu_{out}, I_{in} - \mu_{in})}{\sqrt{\Gamma(I_{out} - \mu_{out}, I_{out} - \mu_{out}) \cdot \Gamma(I_{in} - \mu_{in}, I_{in} - \mu_{in})}} \tag{S4}$$

where the operator Γ is defined as follows: $\Gamma(I_1, I_2) = \sum_{i,j}[I_1(i,j) \cdot I_2(i,j)]$, where i and j are

indices of pixels in the 2D images.

The EPF shows edge preservation effects with respect to the original image that computes the local correlation. These metric are commonly used in OCT denoising studies[3-5], and is defined as follows:

$$EPF = \frac{\sum(\nabla^2 I_{out} - \overline{\nabla^2 I_{out}}) \cdot (\nabla^2 I_{in} - \overline{\nabla^2 I_{in}})}{\sqrt{\sum(\nabla^2 I_{out} - \overline{\nabla^2 I_{out}}) \cdot \sum(\nabla^2 I_{in} - \overline{\nabla^2 I_{in}})}} \tag{S5}$$

where ∇^2 represents the Laplacian operator.

Supplementary Note 2

Imperfect dispersion compensation for datasets of NetA

Dispersion is a wavelength-dependent phenomenon in media, such as the optical components of a system and biological tissues, and can be expressed using phase terms of the acquired raw interference fringes, as follows:

$$\angle I = k(t)d + \theta(t) \tag{S6}$$

where $\angle I$, $k(t)$, d, and $\theta(t)$ are phase of interference fringe, wavenumber according to swept source laser, optical path difference, and dispersion, respectively. Here, $\theta(t)$ can be expressed as a Taylor series expansion for k, as follows[6]:

$$\theta(k) = \theta(k_0) + \left.\frac{\partial\theta(k)}{\partial k}\right|_{k_0}(k_0 - k) + \frac{1}{2}\left.\frac{\partial^2\theta(k)}{\partial k^2}\right|_{k_0}(k_0 - k)^2 + \cdots + \frac{1}{n!}\left.\frac{\partial^n\theta(k)}{\partial k^n}\right|_{k_0}(k_0 - k)^n \tag{S7}$$

In post-processing-based numerical dispersion compensation, equation S6 is linearly approximated as follows to obtain $\theta(t)$, to be subtracted from the phase of the interference signal[7]:

$$\angle\tilde{I} = k(t)d \tag{S8}$$

Then, to approximate $\theta(t)$, we subtract equation S8 from equation S6 to obtain $\widetilde{\theta(t)}$, as follows:

$$\widetilde{\theta(t)} \approx \angle I - \angle\tilde{I} \tag{S9}$$

The numerical dispersion $\widetilde{\theta(t)}$ for phase correction was estimated by fitting Equation S9 to the n-order polynomial; in this study, the numerical dispersion was fitted as a fourth-order polynomial function containing a second-order term describing the group-velocity dispersion. Finally, the following compensation function was estimated by considering the additional dispersion second-order coefficient C to more sophisticatedly compensate for the sample-dependent dispersion that was not sufficiently considered in the above process. Note that the optimal C was estimated empirically.

$$I_\theta = \exp\left(-i(\widetilde{\theta(t)}')\right) \tag{S10}$$

where $\widetilde{\theta(t)}'$ is the result of considering the additional quadratic coefficient correction to the result of fourth-order polynomial fit of equation S9. Here, the input data were randomly selected by adjusting C, representing the additional second-order coefficient correction describing the group-velocity dispersion. The adjustment range was randomly selected from a range where the empirically calculated spatial resolution could only degrade by a maximum of two times. The effects of this are visually represented in **Supplementary Fig.7**.

Supplementary Note 3

Detailed deep learning network architecture for NetA and NetB

In NetA, the generator consists of a total of 13 blocks including four Residual-in-Residual Dense Blocks (RRDB) and 1 skip connection block. RRDB is a structure proposed to replace conventional residual layers; it results in improved computation through continuous memory structures[8]. The skip connection block processes the FFT result and adds it to the middle of the STFT result processing. Each block employs Parametric Rectified Linear Units (PReLU) as an activation function, and 2D batch normalization was applied to accelerate and stabilize training. No final activation function exists. The discriminator consists of 11 blocks based on convolutional layers. Each block employed Leaky-ReLU as an activation function, and 2D batch normalization was applied. Sigmoid with output ranges of 0 and 1 was adapted as the last activation function.

In NetB, the generator consists of a total of 12 blocks, including 8 RRDBs; other details are the same as NetA. The discriminator also differs from NetA only in size of input data; the overall details are the same.

Supplementary Note 4

System implementation

To observe whether overfitting has occurred during training, all of the loss values were monitored and recorded. The training started without early stopping; NetA had a batch size of 12 for 700 epochs and NetB had a batch size of 8 for 700 epochs. When training NetB, the image patch size in the batch was randomly cropped to 256×256. Both models were sequentially optimized using the Adam optimizer, with a learning rate of 0.001[9]. All training methods were implemented using PyTorch (https://pytorch.org/) on a GPU server with four NVIDIA RTX 3090 cards and CUDA V 11.1. Using these hardware specifications, the training took approximately three days for each model. The inference time required to process one B-scan through the two trained models is approximately 1.5 seconds.

Supplementary Tables

Table. S1 Architecture of NetA

Generator

Block #	Block type	Filter Size	Stride	Padding	Number of Filters	Normalization	Activation Function	Output Feature Map Size
1	Conv	3 x 3 x 2	1	1	64		PReLU	14 x 1024 x 64
2	RRDB							14 x 1024 x 64
3	RRDB							14 x 1024 x 64
4	RRDB							14 x 1024 x 64
5	RRDB							14 x 1024 x 64
6	Conv	3 x 3 x 64	1	1	64	Batch 2D	PReLU	14 x 1024 x 64
7	Conv	4 x 3 x 64	1	(0, 1)	64	Batch 2D	PReLU	11 x 1024 x 64
8	Conv	4 x 3 x 64	1	(0, 1)	64	Batch 2D	PReLU	8 x 1024 x 64
9	Conv	4 x 3 x 64	1	(0, 1)	64	Batch 2D	PReLU	5 x 1024 x 64
10	Conv	3 x 3 x 64	1	(0, 1)	64	Batch 2D	PReLU	3 x 1024 x 64
11	Conv	3 x 3 x 64	1	(0, 1)	64	Batch 2D	PReLU	1 x 1024 x 64
12	Conv	3 x 3 x 64	1	1	64	Batch 2D	PReLU	1 x 1024 x 64
	Skip connection	3 x 3 x 2	1	1	64		PReLU	1 x 1024 x 64
13	Conv	3 x 3 x 64	1	1	2			1 x 1024 x 2

RRDB

Block 1	Conv	3 x 3 x 64	1	1	32	Batch 2D	PReLU	14 x 1024 x 32
	Conv	3 x 3 x (64+32)	1	1	32	Batch 2D	PReLU	14 x 1024 x 32
	Conv	3 x 3 x (64+32+32)	1	1	32	Batch 2D	PReLU	14 x 1024 x 32
	Conv	3 x 3 x (64+32+32+32)	1	1	64	Batch 2D	PReLU	14 x 1024 x 64
Block 2	Conv	3 x 3 x 64	1	1	32	Batch 2D	PReLU	14 x 1024 x 32
	Conv	3 x 3 x (64+32)	1	1	32	Batch 2D	PReLU	14 x 1024 x 32
	Conv	3 x 3 x (64+32+32)	1	1	32	Batch 2D	PReLU	14 x 1024 x 32
	Conv	3 x 3 x (64+32+32+32)	1	1	64	Batch 2D	PReLU	14 x 1024 x 64
Block 3	Conv	3 x 3 x 64	1	1	32	Batch 2D	PReLU	14 x 1024 x 32
	Conv	3 x 3 x (64+32)	1	1	32	Batch 2D	PReLU	14 x 1024 x 32
	Conv	3 x 3 x (64+32+32)	1	1	32	Batch 2D	PReLU	14 x 1024 x 32
	Conv	3 x 3 x (64+32+32+32)	1	1	64	Batch 2D	PReLU	14 x 1024 x 64

Discrimiator

1	Conv	3 x 3 x 2	1	1	64		Leaky-ReLU	1 x 1024 x 64
2	Conv	3 x 3 x 64	2	1	64	Batch 2D	Leaky-ReLU	1 x 512 x 128
3	Conv	3 x 3 x 64	1	1	128	Batch 2D	Leaky-ReLU	1 x 512 x 128
4	Conv	3 x 3 x 128	2	1	128	Batch 2D	Leaky-ReLU	1 x 256 x 256
5	Conv	3 x 3 x 128	1	1	256	Batch 2D	Leaky-ReLU	1 x 256 x 256
6	Conv	3 x 3 x 256	2	1	256	Batch 2D	Leaky-ReLU	1 x 128 x 256
7	Conv	3 x 3 x 256	1	1	512	Batch 2D	Leaky-ReLU	1 x 128 x 512
8	Conv	3 x 3 x 512	2	1	512	Batch 2D	Leaky-ReLU	1 x 64 x 512

9	Conv	3 x 3 x 512	1	1	512	Batch 2D	Leaky-ReLU	1 x 64 x 512
10	Conv	3 x 3 x 512	2	1	512	Batch 2D	Leaky-ReLU	1 x 32 x 512
11	FCN						Leaky-ReLU	1 x 1 x 16384
12	FCN							1 x 1 x 1

Table. S2 Architecture of NetB

Generator

Block #	Block type	Filter Size	Stride	Padding	Number of Filters	Normalization	Activation Function	Output Feature Map Size
1	Conv	3 x 3 x 1	1	1	64		PReLU	256 x 256 x 64
2	RRDB							256 x 256 x 64
3	RRDB							256 x 256 x 64
4	RRDB							256 x 256 x 64
5	RRDB							256 x 256 x 64
6	RRDB							256 x 256 x 64
7	RRDB							256 x 256 x 64
8	RRDB							256 x 256 x 64
9	RRDB							256 x 256 x 64
10	Conv	3 x 3 x 64	1	1	64	Batch 2D	PReLU	256 x 256 x 64
11	Conv	3 x 3 x 64	1	1	64	Batch 2D	PReLU	256 x 256 x 64
12	Conv	3 x 3 x 64	1	1	64	Batch 2D	PReLU	256 x 256 x 64
13	Conv	3 x 3 x 64	1	1	1			256 x 256 x 1

Discriminator

RRDB								
Block 1	Conv	3 x 3 x 64	1	1	32	Batch 2D	PReLU	256 x 256 x 32
	Conv	3 x 3 x (64+32)	1	1	32	Batch 2D	PReLU	256 x 256 x 32
	Conv	3 x 3 x (64+32+32)	1	1	32	Batch 2D	PReLU	256 x 256 x 32
	Conv	3 x 3 x (64+32+32+32)	1	1	64	Batch 2D	PReLU	256 x 256 x 64
Block 2	Conv	3 x 3 x 64	1	1	32	Batch 2D	PReLU	256 x 256 x 32
	Conv	3 x 3 x (64+32)	1	1	32	Batch 2D	PReLU	256 x 256 x 32
	Conv	3 x 3 x (64+32+32)	1	1	32	Batch 2D	PReLU	256 x 256 x 32
	Conv	3 x 3 x (64+32+32+32)	1	1	64	Batch 2D	PReLU	256 x 256 x 64
Block 3	Conv	3 x 3 x 64	1	1	32	Batch 2D	PReLU	256 x 256 x 32
	Conv	3 x 3 x (64+32)	1	1	32	Batch 2D	PReLU	256 x 256 x 32
	Conv	3 x 3 x (64+32+32)	1	1	32	Batch 2D	PReLU	256 x 256 x 32
	Conv	3 x 3 x (64+32+32+32)	1	1	64	Batch 2D	PReLU	256 x 256 x 64
1	Conv	3 x 3 x 1	1	1	64		Leaky-ReLU	256 x 256 x 64
2	Conv	3 x 3 x 64	2	1	64	Batch 2D	Leaky-ReLU	128 x 128 x 128
3	Conv	3 x 3 x 64	1	1	128	Batch 2D	Leaky-ReLU	128 x 128 x 128
4	Conv	3 x 3 x 128	2	1	128	Batch 2D	Leaky-ReLU	64 x 64 x 256
5	Conv	3 x 3 x 128	1	1	256	Batch 2D	Leaky-ReLU	64 x 64 x 256
6	Conv	3 x 3 x 256	2	1	256	Batch 2D	Leaky-ReLU	32 x 32 x 256
7	Conv	3 x 3 x 256	1	1	512	Batch 2D	Leaky-ReLU	32 x 32 x 512
8	Conv	3 x 3 x 512	2	1	512	Batch 2D	Leaky-ReLU	16 x 16 x 512
9	Conv	3 x 3 x 512	1	1	512	Batch 2D	Leaky-ReLU	16 x 16 x 512
10	Conv	3 x 3 x 512	2	1	512	Batch 2D	Leaky-ReLU	8 x 8 x 512

11	FCN		Leaky-ReLU	1 x 1 x 32768
12	FCN			1 x 1 x 1

Supplementary Figures

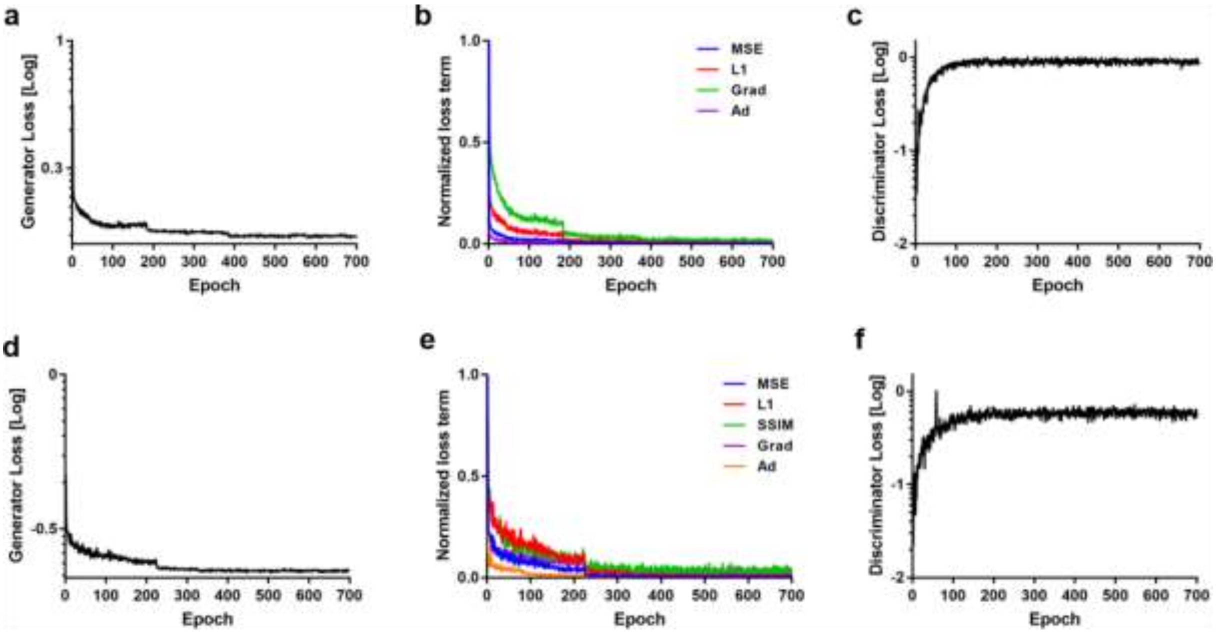

Figure S1. Training loss curve. a-c represent plots of training loss of proposed NetA in training for each epoch**. a** represents plot of training loss for the generator. **b** represents normalized plot of each term included in **a**. **c** represents plot of training loss for discriminator on log compressed scale. **d-f** represent plots of training loss of proposed NetB in training for each epoch. **d** and **e** represent plots of training loss for generator and normalized plot of each term included in generator loss, respectively. **f** represents plot of training loss for discriminator on log compressed scale. The generator losses in **a** and **d** gradually converge, revealing that the results of NetA and NetB are similar to the ground truth and can easily deceive the discriminator. In contrast, the discriminator losses in **c** and **f** gradually increase, indicating that the results of the generator are indistinguishable from the ground truth.

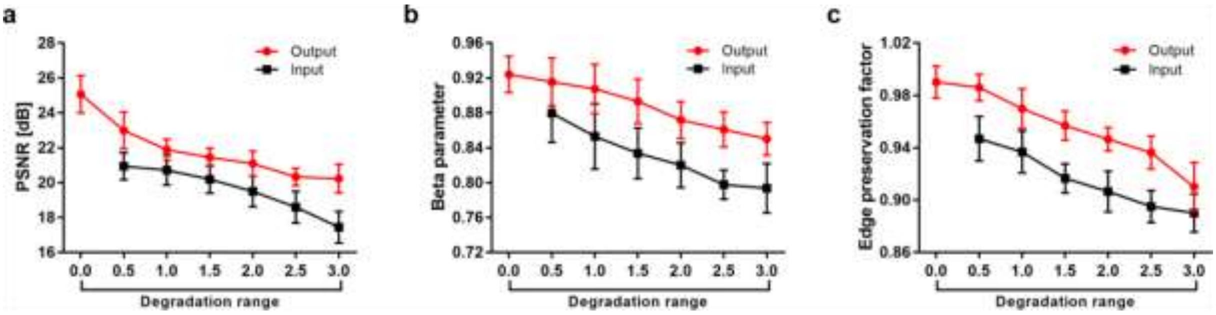

Figure S2. Performance test of deep learning-based OCT image enhancement framework according to the degree of input data degradation. a PSNR, **b** β, and **c** EPF evaluation results of inputs and outputs according to the degree of degradation. In all graphs, the x-axis denotes the relative degree of degradation, where "0.0" corresponds to the input with no degradation, i.e., equivalent to the ground truth (currently optimized OCT data). This test evaluates the quality of the degraded input and corresponding output against ground truth, presenting a comparative representation of the extent of performance improvement against the degree of degradation. Note that quantitative evaluation is not conducted on the input when the degradation degree is "0.0" because it is the same as the ground truth. The results show that the presented method can produce enhanced images when the input is either undegraded (currently optimized OCT image) or when the level of degradation is low. The findings also demonstrate that, even images with higher levels of degradation can be restored to comparable performance levels to the original OCT image. All error bars represent standard deviations. All of these statistical results were calculated for 200 randomly selected images.

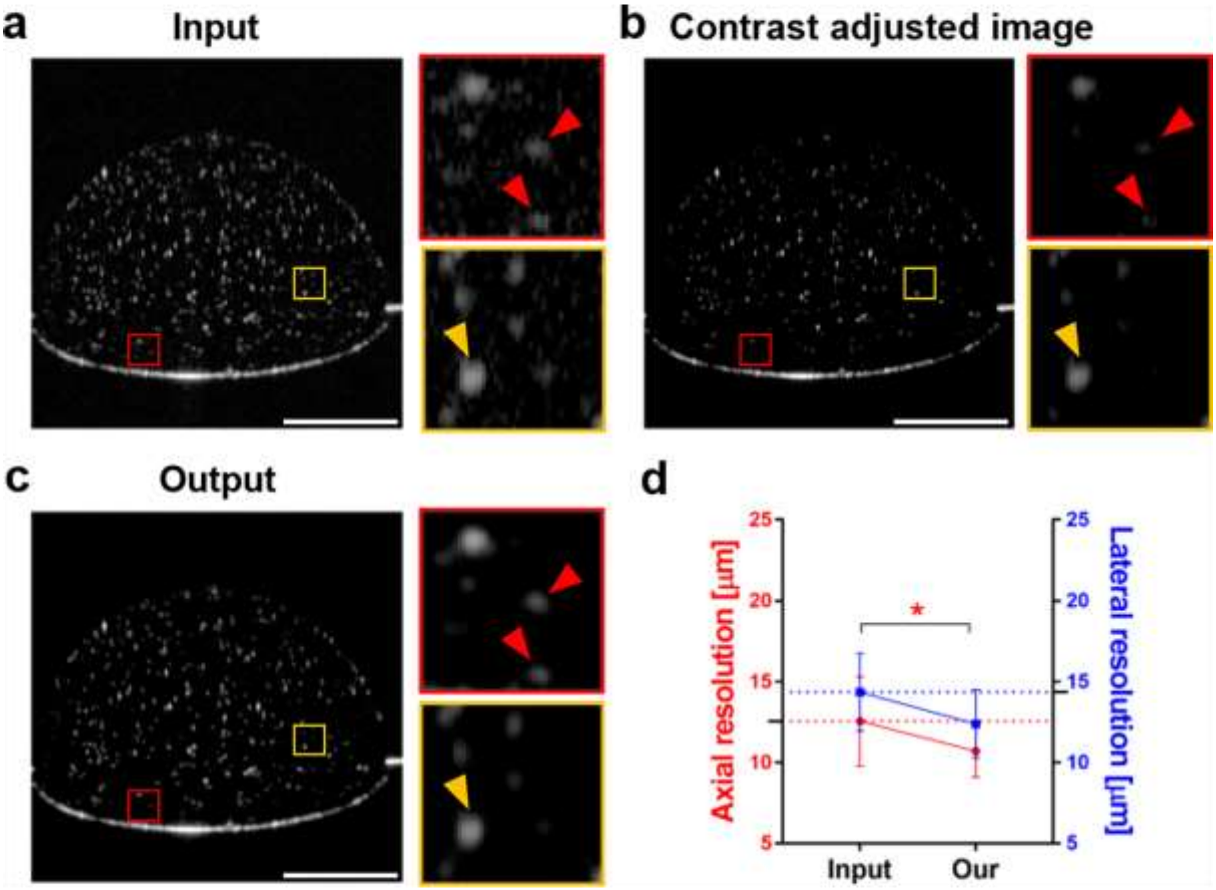

Figure S3. Comparison of spatial resolution enhancement with contrast-adjusted image. a Image of TiO2 microspheres. **b** Contrast-adjusted image of **a**. **c** Output of processing **a** with the proposed deep learning-based framework. **b** was constructed by adjusting the intensity level and background level of **a** to be similar to that of **c**. **d** Axial and lateral resolution measured with data from **a** and **c**. The spatial resolution appears to be enhanced when the contrast is adjusted (yellow arrowheads in **b**). However, the contrast adjustment causes the weak signal to disappear excessively (red arrowheads in **b**), resulting in a large loss of spatial information. Therefore, this seemingly enhancement of resolution is not true. Whereas, in the output of deep learning (**c**), the spatial resolution is enhanced for microspheres (yellow arrowheads in **c**) even for weak signal (red arrowheads in **c**). Moreover, the degree of our output enhancement is also far superior. The ROIs (red and yellow boxes) on the right side of the image show magnified views (5X). Scale bars, 1 mm. Error bars in **d** represent standard deviations. In **d**, comparison results were statistically significant ($P<0.0001$ (*) according to a one-way ANOVA test). These statistical results were calculated for 200 randomly selected microspheres.

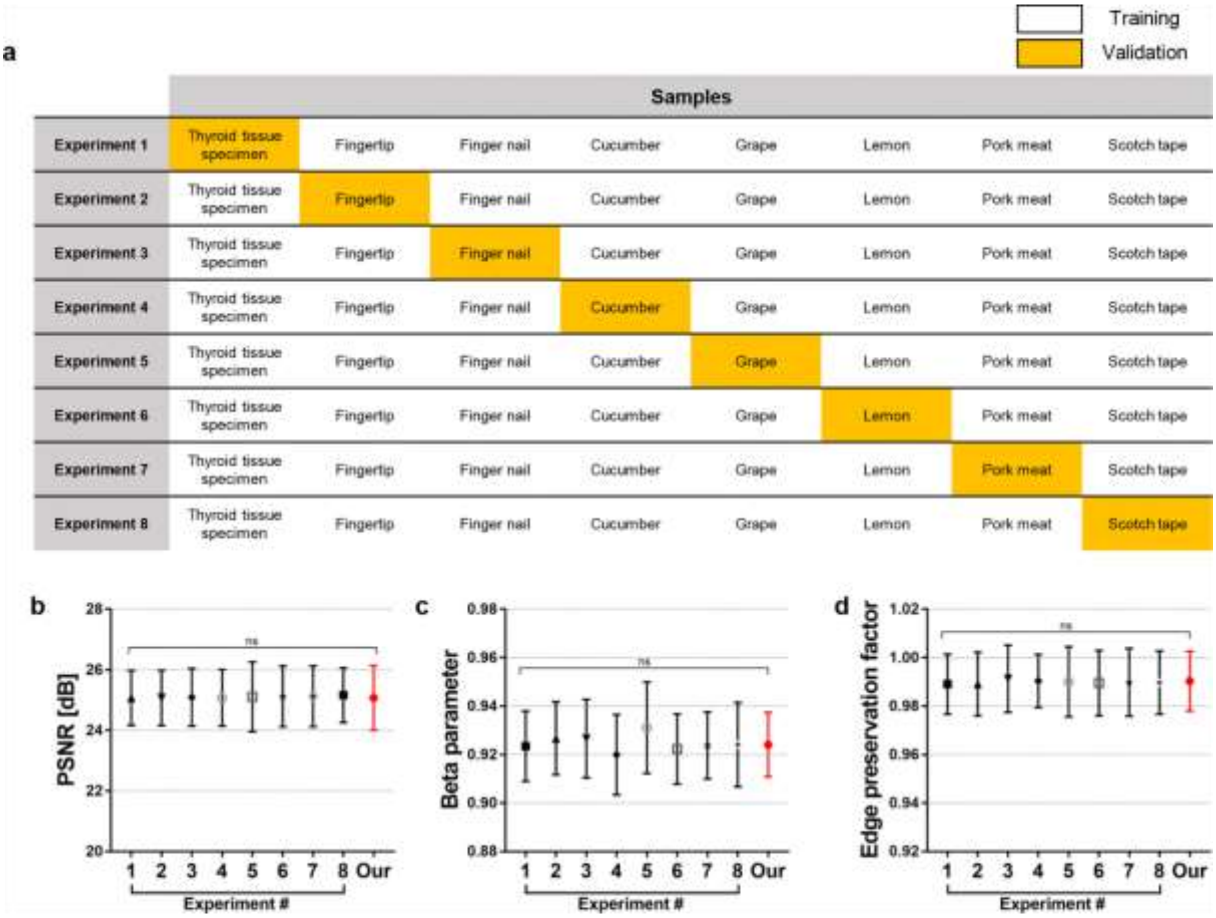

Figure S4 Performance evaluation result through multi-fold analysis. a Dataset configuration for multi-fold analysis**.** One of the eight samples served as the validation set, and others served as the training set, configuring up a total of eight datasets. **b-d** Quantitative evaluation results measured by **b** PSNR, **c** Beta parameter (β), and **d** EPF. The x-axis of each result displays the experiment number for each dataset as well as the original assessed result denoted by "our". All metrics showed outcomes that are comparable to those determined by the values assessed by the original trained model. This demonstrates that our model was successfully trained without overfitting. Error bars in **b-d** represent standard deviations. All of these statistical results were calculated for 200 randomly selected images. In **b-d**, all multiple comparison results were statistically nonsignificant (P>0.999 (ns) according to a one-way ANOVA test).

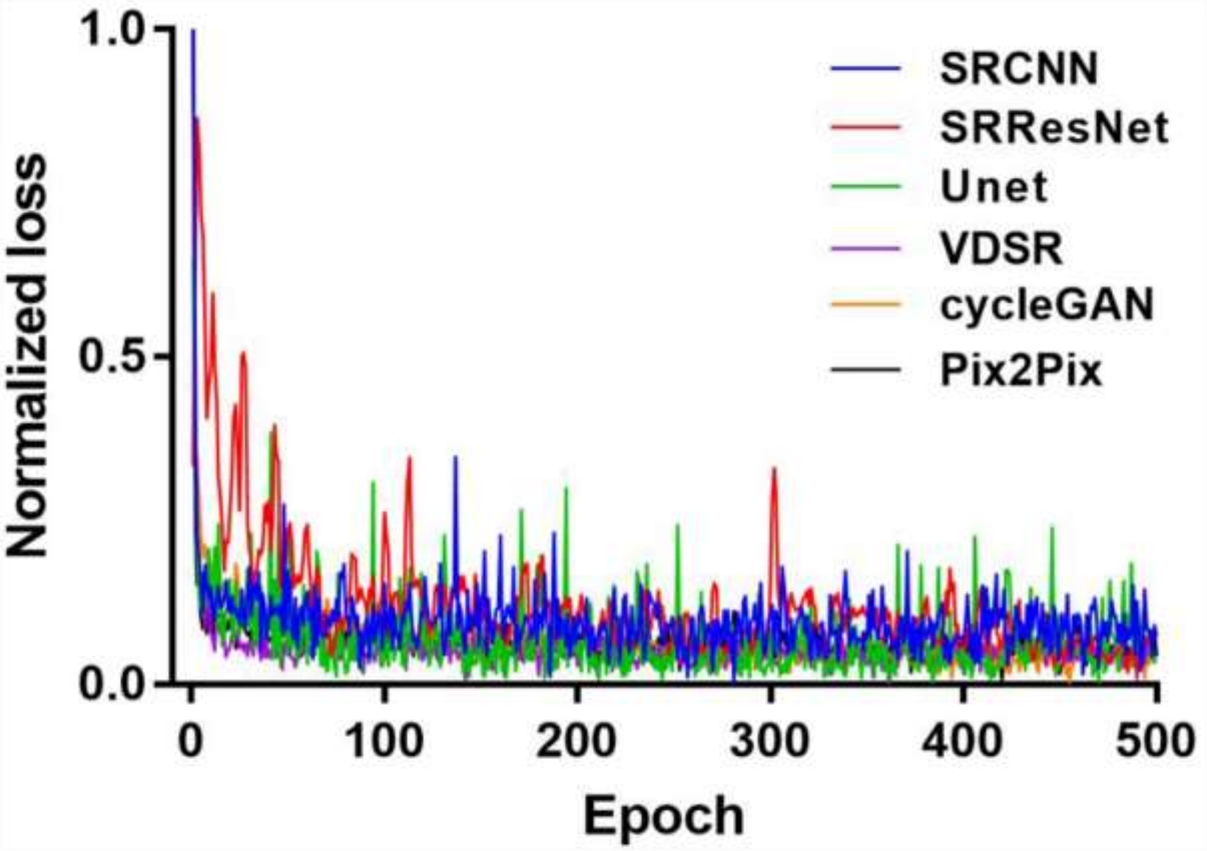

Figure S5. Training loss curves of other deep learning techniques for comparative studies. This figure represents training loss plots of deep learning techniques (SRCNN, SRResNet, Unet, VDSR, cycleGAN, and Pix2Pix) retrained for comparative studies. The loss function of each techniques was adopted as in the literature, and each plot was normalized. Losses gradually converged for all techniques, indicating successful training.

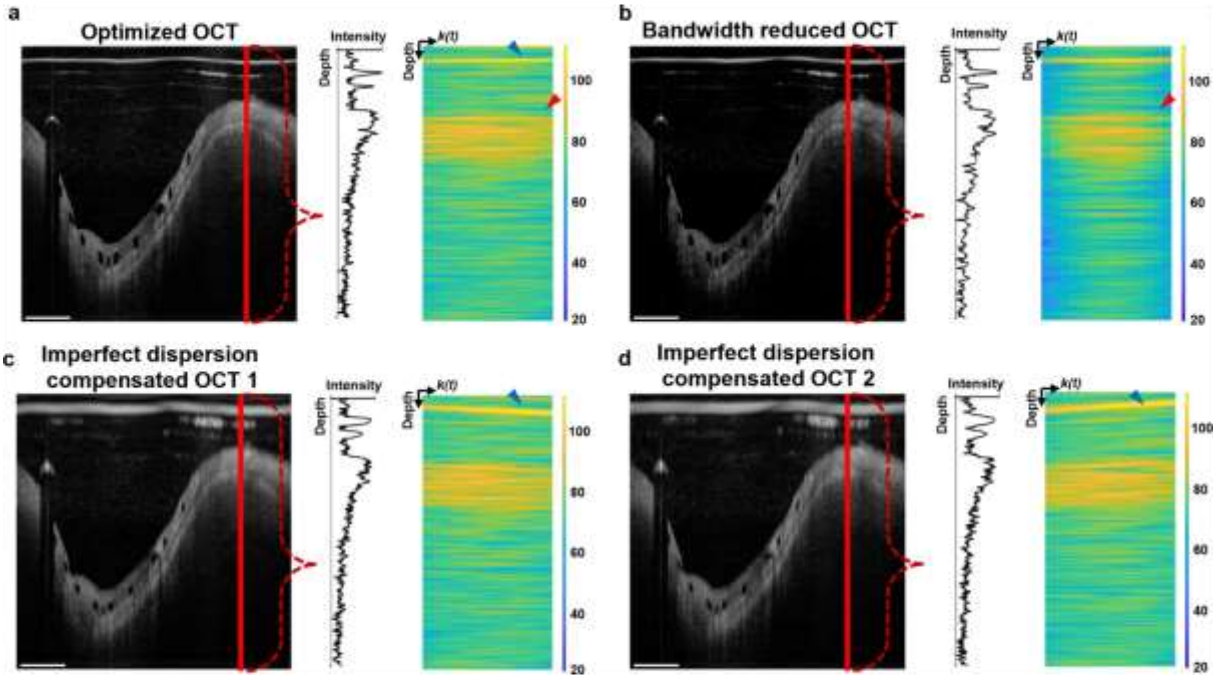

Figure S6. Examples of OCT images and spectrograms. In **a-d**, the left is the B-scan, and the right is the A-scan of one of the samples and the corresponding spectrogram. **a** Results for currently optimized OCT data. **b** Results for bandwidth truncated OCT data. **c-d** Results for OCT data with imperfect dispersion compensation. In **a** and **b**, the red arrows clearly indicate differences in spectral information due to truncating bandwidth. Specifically, it is revealed that the intensity decreases as it approaches the truncated bandwidth region in the horizontal axis, which represents the wavenumber domain. In **a**, **c**, and **d**, the blue arrows show differences in spectral information according to the dispersion compensation. This arrows indicate that the depth-dependent spectral information does not appear stable in terms of k due to imperfect dispersion compensation. Scale bars, 1 mm.

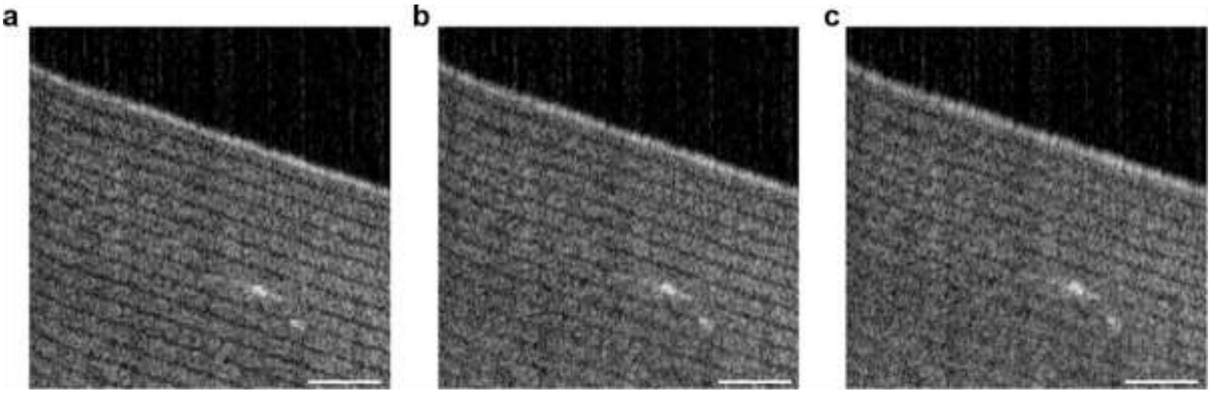

Figure S7. Examples of OCT images according to dispersion compensation. This figures show results of OCT image degradation according to post-processing based numerical dispersion compensation. **a** Image of maximum achievable axial resolution obtained by currently optimized dispersion compensation. **b-c** Image of reduced axial resolution by imperfect dispersion compensation using same interference fringes as **a**. The degradation of the axial resolution can be visually recognized. **c** shows results of greatest degradation in axial resolution by maximally adjusting second-order term coefficient within predefined range. **b** is the result of adjusting to half the level of **c**. Scale bars, 300 μm.

References

1 Adler, D. C., Ko, T. H. & Fujimoto, J. G. Speckle reduction in optical coherence tomography images by use of a spatially adaptive wavelet filter. *Optics letters* **29**, 2878-2880 (2004).

2 Zaki, F., Wang, Y., Su, H., Yuan, X. & Liu, X. Noise adaptive wavelet thresholding for speckle noise removal in optical coherence tomography. *Biomedical optics express* **8**, 2720-2731 (2017).

3 Wong, A., Mishra, A., Bizheva, K. & Clausi, D. A. General Bayesian estimation for speckle noise reduction in optical coherence tomography retinal imagery. *Optics express* **18**, 8338-8352 (2010).

4 Gong, G., Zhang, H. & Yao, M. Speckle noise reduction algorithm with total variation regularization in optical coherence tomography. *Optics express* **23**, 24699-24712 (2015).

5 Li, M., Idoughi, R., Choudhury, B. & Heidrich, W. Statistical model for OCT image denoising. *Biomedical optics express* **8**, 3903-3917 (2017).

6 Drexler, W. & Fujimoto, J. G. *Optical coherence tomography: technology and applications*. Vol. 2 (Springer, 2015).

7 Wojtkowski, M. *et al.* Ultrahigh-resolution, high-speed, Fourier domain optical coherence tomography and methods for dispersion compensation. *Optics express* **12**, 2404-2422 (2004).

8 Wang, X. *et al.* in *Proceedings of the European conference on computer vision (ECCV) workshops.* 0-0.

9 Kingma, D. P. & Ba, J. Adam: A method for stochastic optimization. *arXiv preprint arXiv:1412.6980* (2014).
