# Supplementary material for: Deep learning-based image enhancement in optical coherence tomography by exploiting interference fringe
Source: Commun Biol. 2023 Apr 28;6:464. doi: 10.1038/s42003-023-04846-7 (PMC10147647; doi:10.1038/s42003-023-04846-7)
Supplement: Supplementary file 3 — Description of Additional Supplementary Files [file 42003_2023_4846_MOESM3_ESM.pdf]

Description of Additional Supplementary Files

File name: Supplementary Data 1
Description: The source data presented in the main figures.
