# Supplementary material for: Deep learning-based image enhancement in optical coherence tomography by exploiting interference fringe
Source: Commun Biol. 2023 Apr 28;6:464. doi: 10.1038/s42003-023-04846-7 (PMC10147647; doi:10.1038/s42003-023-04846-7)
Supplement: Supplementary file 5 — Reporting Summary [file 42003_2023_4846_MOESM5_ESM.pdf]

nature portfolio

Corresponding author(s): Prof. Dr. Hongki Yoo

Last updated by author(s): Apr 6, 2023

Reporting Summary

Nature Portfolio wishes to improve the reproducibility of the work that we publish. This form provides structure for consistency and transparency in reporting. For further information on Nature Portfolio policies, see our Editorial Policies and the Editorial Policy Checklist.

Statistics

For all statistical analyses, confirm that the following items are present in the figure legend, table legend, main text, or Methods section.

n/a	Confirmed	
☐	☒	The exact sample size (*n*) for each experimental group/condition, given as a discrete number and unit of measurement
☐	☒	A statement on whether measurements were taken from distinct samples or whether the same sample was measured repeatedly
☐	☒	The statistical test(s) used AND whether they are one- or two-sided<br>*Only common tests should be described solely by name; describe more complex techniques in the Methods section.*
☒	☐	A description of all covariates tested
☒	☐	A description of any assumptions or corrections, such as tests of normality and adjustment for multiple comparisons
☐	☒	A full description of the statistical parameters including central tendency (e.g. means) or other basic estimates (e.g. regression coefficient) AND variation (e.g. standard deviation) or associated estimates of uncertainty (e.g. confidence intervals)
☒	☐	For null hypothesis testing, the test statistic (e.g. *F*, *t*, *r*) with confidence intervals, effect sizes, degrees of freedom and *P* value noted<br>*Give P values as exact values whenever suitable.*
☒	☐	For Bayesian analysis, information on the choice of priors and Markov chain Monte Carlo settings
☒	☐	For hierarchical and complex designs, identification of the appropriate level for tests and full reporting of outcomes
☒	☐	Estimates of effect sizes (e.g. Cohen's *d*, Pearson's *r*), indicating how they were calculated

Our web collection on statistics for biologists contains articles on many of the points above.

Software and code

Policy information about availability of computer code

Data collection	Data were collected using a previously reported benchtop-based and catheter-based SS-OCT (detailed informations can be found in the "Materials and Methods" section). The dataset obtained from benchtop-based SS-OCT system consists of a total of 12 samples (5 thyroid tissue specimens, fingernails, fingertips, cucumber, grape, lemon, pork, and Scoth tape). The dataset acquired with the other system consists of a total of 35 pullback sets, of which 23 were from in vivo swine coronary arteries implanted with bioresorbable scaffolds; the remaining 12 were from rabbit abdominal aortas with atherosclerotic plaque. All pullback sets were obtained in different regions of each vessel or in different animals.
Data analysis	Preprocessing of data for preparation of training datasets was performed using the code in Matlab R2021a. All training methods were implemented using PyTorch (https://pytorch.org/) on a GPU server with four NVIDIA RTX 3090 cards and CUDA V 11.1. Deep learning-based OCT signal processing framework can be found in the oct_enhancement_framework repository (https://github.com/KAIST-BOOM/oct-enhancement-framework)

For manuscripts utilizing custom algorithms or software that are central to the research but not yet described in published literature, software must be made available to editors and reviewers. We strongly encourage code deposition in a community repository (e.g. GitHub). See the Nature Portfolio guidelines for submitting code & software for further information.

Data

Policy information about availability of data

All manuscripts must include a data availability statement. This statement should provide the following information, where applicable:

- Accession codes, unique identifiers, or web links for publicly available datasets
- A description of any restrictions on data availability
- For clinical datasets or third party data, please ensure that the statement adheres to our policy

All the source data presented in the main figures are available as Supplementary Data 1. The imaging datasets generated and/or analysed during the current study are available from the corresponding author on reasonable request.

Human research participants

Policy information about studies involving human research participants and Sex and Gender in Research.

Reporting on sex and gender	N/A
Population characteristics	N/A
Recruitment	N/A
Ethics oversight	N/A

Note that full information on the approval of the study protocol must also be provided in the manuscript.

Field-specific reporting

Please select the one below that is the best fit for your research. If you are not sure, read the appropriate sections before making your selection.

☒ Life sciences ☐ Behavioural & social sciences ☐ Ecological, evolutionary & environmental sciences

For a reference copy of the document with all sections, see nature.com/documents/nr-reporting-summary-flat.pdf

Life sciences study design

All studies must disclose on these points even when the disclosure is negative.

Sample size	The acquired training data consists of a total of 12 samples (5 different thyroid tissue specimens, finger nails, fingertip, cucumber, grape, lemon, pork meat, and Scoth tape). For each sample, a total of 5 sets were obtained in different regions. Each set consists of 1,000 B-scans consisting of 1,024 A-scans with a depth of 2,048 pixels; thus, the total number of B-scans in the dataset is 60,000.
Data exclusions	No data was excluded from statistical analysis.
Replication	Results obtained were highly reproducible, obtaining very similar performance in several independent inferences.
Randomization	Test dataset were randomly separated from training dataset. Randomization of data during the training of the deep learning networks was automatically performed.
Blinding	No blinding was required for this study.

Reporting for specific materials, systems and methods

We require information from authors about some types of materials, experimental systems and methods used in many studies. Here, indicate whether each material, system or method listed is relevant to your study. If you are not sure if a list item applies to your research, read the appropriate section before selecting a response.

Materials & experimental systems

n/a	Involved in the study
☒	☐ Antibodies
☒	☐ Eukaryotic cell lines
☒	☐ Palaeontology and archaeology
☐	☒ Animals and other organisms
☒	☐ Clinical data
☒	☐ Dual use research of concern

Methods

n/a	Involved in the study
☒	☐ ChIP-seq
☒	☐ Flow cytometry
☒	☐ MRI-based neuroimaging

Animals and other research organisms

Policy information about studies involving animals; ARRIVE guidelines recommended for reporting animal research, and Sex and Gender in Research

Laboratory animals	A male Yucatan minipigs weighting approximately 15-20kg(Optipharm, Korea). A male New Zealand white rabbit weighting approximately 3-3.5kg (DooYeol Biotech, Korea).
Wild animals	N/A
Reporting on sex	No sex considerations were required in this study.
Field-collected samples	N/A
Ethics oversight	All animal experiments were approved by the Institutional Animal Care and Use Committee of Korea University (KOREA-2019-0152-C1, KOREA-2021-0076) and were performed in accordance with national and institutional guidelines.

Note that full information on the approval of the study protocol must also be provided in the manuscript.
